# Acceso a anticoncepción en adolescentes: percepciones de trabajadores de la salud en Huechuraba, Chile

**DOI:** 10.26633/RPSP.2017.77

**Published:** 2017-04-28

**Authors:** Giovanna Rojas Ramírez, Pamela Eguiguren Bravo, María Isabel Matamala Vivaldi, Irma Palma Manríquez, y Thelma Gálvez Pérez

**Affiliations:** * En este manuscrito se emplean las expresiones “los hijos” En este manuscrito se emplean las expresiones “los hijos” En este manuscrito se emplean las expresiones “los hijos”, “las matronas”, “las enfermeras”, “los adolescentes”, “los jóvenes”, “los técnicos”, etc. de manera inclusiva para denotar ambos sexos, excepto que se aclare lo contrario.; 1 Departamento de Salud Municipalidad de Huechuraba Chile Chile Departamento de Salud, Municipalidad de Huechuraba, Chile.; 2 Escuela de Salud Pública Dr. Salvador Allende Facultad de Medicina, Universidad de Chile Chile Escuela de Salud Pública Dr. Salvador Allende, Facultad de Medicina, Universidad de Chile, Chile.; 3 Observatorio de Equidad de Género en Salud Observatorio de Equidad de Género en Salud Chile Chile Observatorio de Equidad de Género en Salud, Chile.; 4 Facultad de Ciencias Sociales Universidad de Chile Chile Chile Facultad de Ciencias Sociales, Universidad de Chile, Chile.

**Keywords:** Anticoncepción, adolescente, embarazo en adolescencia, derechos sexuales y reproductivos, Chile, Contraception, adolescent, pregnancy in adolescence, sexual and reproductive rights, Chile, Anticoncepção, adolescente, gravidez na adolescência, direitos sexuais e reprodutivos, Chile

## Abstract

**Objetivo.:**

*Identificar dificultades para el acceso a atención e información en anticoncepción de adolescentes desde percepciones y experiencias de trabajadores de la salud de Huechuraba, en la Región Metropolitana de Chile*.

**Métodos.:**

*Este estudio cualitativo y descriptivo incorporó principios de investigación acción participativa involucrando a equipos de atención en el levantamiento y análisis de información, con generación de propuestas de mejora. Se realizaron 17 entrevistas individuales semiestructuradas y una entrevista grupal, con profesionales y técnicos involucrados en la atención de adolescentes en centros de salud de la comuna*.

**Resultados.:**

*Trabajadores de la salud percibían dificultades en la llegada de adolescentes a los centros por razones relacionadas a factores culturales, falta de información y de actividades de salud en la comunidad. Existen requisitos administrativos y tramitaciones que obstaculizan el acceso a la atención. Se evidenciaron falencias en el manejo e interpretación de normas de regulación de la fertilidad y de la legislación vigente y ausencia de marcos explicativos que reconociesen el género y derechos sexuales y reproductivos de los adolescentes*.

**Conclusiones.:**

*Existe poca visibilidad de los adolescentes y sus necesidades, y contradicciones entre los discursos y las prácticas, con ausencia de definiciones y acuerdos para el acceso a anticoncepción y consejería que consideren contextos sociales y culturales. Urge la implementación de acciones de capacitación para trabajadores de la salud en género y derechos sexuales y reproductivos, junto con espacios de reflexión para generar abordajes articulados y efectivos. Se requieren esfuerzos de difusión del programa y realización de actividades en espacios comunitarios, junto con otros sectores comunales*.

## Abstract (en)

**Objective.:**

Identify difficulties in adolescents’ access to contraceptive care and information, based on perceptions and experiences of health workers in Huechuraba, in the Metropolitan Region of Chile.

**Methods.:**

This qualitative, descriptive study incorporated principles of participatory action research, involving health care teams in the survey and data analysis, and generating proposals for improvement. Seventeen (17) semi-structured individual interviews and one group interview were conducted with professionals and technical personnel involved in adolescent care in the commune’s health centers.

**Results.:**

Health workers perceived that adolescents were having difficulties reaching the centers due to cultural factors, lack of information, lack of health activities in the community, while administrative requirements and procedures hindered access to care. Shortcomings were evident in the management and interpretation of fertility regulation standards and of current legislation, and in the absence of explanatory frameworks recognizing adolescents’ gender, sexual, and reproductive rights.

**Conclusions.:**

Adolescents and their needs have a low profile and there are conflicts between theory and practice regarding access to contraception and counseling, with a lack of definitions or agreements that take into account social and cultural contexts. It is urgent to provide health workers with training on gender and sexual and reproductive rights, together with opportunities for reflection in order to generate coordinated and effective approaches. Efforts are required to disseminate the program and organize activities in community spaces, together with other community sectors.

## RESUMO

**Objetivo.:**

Identificar dificuldades de acesso à atenção e informações relacionadas aos métodos anticoncepcionais entre adolescentes com base em percepções e experiências dos profissionais de saúde de Huechuraba, na região metropolitana de Santiago, Chile.

**Métodos.:**

Estudo qualitativo descritivo realizado segundo os princípios de pesquisaação participativa envolvendo pessoal da saúde na coleta e análise das informações, com a produção de propostas para melhoria. Foram realizadas 17 entrevistas semiestruturadas individuais e uma entrevista em grupo com profissionais e pessoal técnico que prestam atenção a adolescentes em centros de saúde comunitários.

**Resultados.:**

Os profissionais de saúde perceberam dificuldade na chegada dos adolescentes aos centros por fatores culturais e pela falta de informação e de atividades de saúde na comunidade. Existem requisitos administrativos e diligências que são obstáculos ao acesso à atenção. Foram evidenciadas falhas na gestão e interpretação das normas de regulação da fertilidade e da legislação vigente e a ausência de enquadramentos explicativos que reconhecessem o gênero e os direitos sexuais e reprodutivos dos adolescentes.

**Conclusões.:**

Os adolescentes e suas necessidades têm pouca visibilidade e existem contradições entre os discursos e as práticas, com a falta de definições e acordos para o acesso a métodos anticoncepcionais e orientação que levam em consideração os contextos socioculturais. É premente a implementação de ações de capacitação para os profissionais de saúde em gênero e direitos sexuais e reprodutivos, assim como de espaços de reflexão para elaborar enfoques articulados e efetivos. São necessários esforços para divulgar o programa e realizar atividades em espaços comunitários, junto com outros setores da comunidade.

Del total de nacidos vivos durante el año 2012 en Chile, un 14,4% fueron hijos* de madres adolescentes y, ese año, cerca de 900 de esos nacimientos correspondieron a madres niñas menores de 15 años (1). La tasa de fecundidad adolescente en 2012 fue de 26,14 por mil mujeres entre 10 y 19 años y de 48,6 por mil entre 15 y 19 años. Aunque las cifras nos ubican mejor que un conjunto importante de países de la región (2, 3), y han descendido respecto de años anteriores, detrás de esta imagen optimista se esconden grandes desigualdades. En 2010, entre la comuna más pobre y la más rica de la Región Metropolitana, se observó una diferencia de 18,6 puntos porcentuales en la proporción de nacidos de mujeres adolescentes (La Pintana, 20,9% frente a Las Condes, 2,3%) (1). Es un hecho reconocido que el embarazo adolescente actúa como un mecanismo reproductor de inequidades de género y aumenta la vulnerabilidad de las mujeres (4, 5). La magnitud e implicancias sociosanitarias de este problema explican su inclusión en los Objetivos Sanitarios de la Década 2011-2020 para Chile, en los que se procura la disminución de la tasa de fecundidad en la población menor de 19 años, con estrategias orientadas a mejorar el acceso a servicios y a prestaciones en materia de salud sexual y reproductiva (6).

En 2010 se promulgó la Ley No. 20 418 (7)que fijó normas sobre información, orientación y prestaciones en materia de regulación de fertilidad. La ley indica, en su artículo 1º, que “toda persona —desde los 14 años— tiene derecho a recibir educación, información y orientación en materia de regulación de la fertilidad, en forma clara, comprensible, completa y, en su caso, confidencial”. El Programa Nacional de Salud Integral de Adolescentes y Jóvenes (8) busca mejorar la salud y desarrollo de los adolescentes mediante una atención multidisciplinaria e intervenciones integradas. A pesar de estos avances, el acceso a consejería y anticoncepción de adolescentes enfrenta múltiples dificultades para su implementación a nivel local. Diversos estudios nacionales señalan que profesionales y funcionarios del sistema público con frecuencia carecen de información sobre género y derechos sexuales y reproductivos y sobre el contenido de la Ley y normas de regulación de la fertilidad, y destacan como obstáculos la existencia de prejuicios respecto de la autonomía de los adolescentes y la falta de espacios diferenciados para la atención pertinente a esta población (9, 10).

Investigaciones sobre barreras y facilitadores en el acceso a la salud sexual y reproductiva de adolescentes a nivel internacional y en la Región muestran de forma coincidente que las principales dificultades están focalizadas en la falta de competencias de los profesionales que atienden a adolescentes y jóvenes (11-17). Con respecto a la calidad de la atención brindada, destaca la ausencia de trato empático y amigable, y la atención en horarios que faciliten el acceso de los adolescentes, señalando también la falta de espacios que garanticen la confidencialidad (13, 14, 16, 18-20). Los resultados de una evaluación cualitativa de programas en Argentina, Brasil y México publicados por el Consorcio Latinoamericano de Programas en Salud Sexual y Reproductiva y Sexualidad han constatado la falta de enfoque de género y el reconocimiento de los derechos sexuales y reproductivos de los adolescentes por parte de profesionales (21) y agregan la escasa ejecución de actividades de educación y prevención que tienen lugar en la comunidad.

No obstante, el conocimiento desarrollado en este tema y el hecho de tener claras las barreras de acceso detectadas, los estudios mencionados comparten la necesidad de mayor investigación en esta área (11, 18, 22, 23) y profundización a través de métodos cualitativos (14). El paradigma cualitativo de investigación, en particular desde la perspectiva fenomenológica, reconoce en sus métodos de producción de conocimiento la indagación de la propia experiencia. Por otra parte, este mismo paradigma sustenta la investigación acción como método, donde se agregan evidencias acerca de que los procesos que generan conocimiento y reflexión desde los propios equipos y efectores de programas tienen mejores posibilidades de gatillar propuestas efectivas de mejora (24).

La investigación que origina este artículo se realizó en Huechuraba, comuna situada en la Región Metropolitana, en Santiago de Chile. Esta comuna tenía una población inscrita para el año 2015 de 11 020 adolescentes (5 562 varones y 5 458 mujeres). En diciembre de ese año, 950 adolescentes mujeres se encontraban bajo control por anticoncepción y en el año se registraron 606 consejerías y se entregaron solo 17 anticonceptivos de emergencia. En forma paralela, la comuna ingresó a 133 mujeres adolescentes al control de embarazo en sus centros de atención, dos de ellas menores de 15 años. Considerando que, según estudios nacionales, 50% de los adolescentes inician su actividad sexual entre los 15 y 19 años, estas cifras resultan muy preocupantes. Frente a ello cabe preguntarse cuáles son los problemas, obstáculos y deficiencias de los servicios de atención primaria de la comuna de Huechuraba que limitan el acceso a consejería y anticoncepción de los adolescentes. El objetivo del presente artículo da cuenta de los resultados de una primera indagación, que buscó identificar la ruta y dificultades de acceso de adolescentes a anticoncepción desde la perspectiva de los trabajadores de la salud. Se encuentra en desarrollo una segunda fase de investigación con los propios adolescentes.

## MÉTODOS

Este trabajo forma parte de la iniciativa: “Mejoras en la ejecución de programas a través de investigaciones acerca de dicha ejecución integrada (iPIER)”, desarrollado por la Organización Panamericana de la Salud (OPS) en colaboración con la Alianza para la Investigación en Políticas y Sistemas de Salud (AHPSR). El modelo iPIER coloca a los ejecutores de programas en el centro de una investigación con el objetivo de entender las fallas en los sistemas de salud que crean barreras a la implementación, así como permite identificar las soluciones a estas. Las investigaciones sobre la ejecución integradas en los procesos existentes apoyan la efectividad de los programas y políticas de salud eficaces a través de la utilización de la investigación como parte del proceso de implementación. Una descripción detallada de la aplicación de la metodología de investigación se incluye en el documento conceptual iPIER (25).

El equipo de trabajo fue conformado por la referente técnica del Departamento de Salud de la Comuna de Huechuraba e investigadoras del Observatorio de Equidad y Género en Salud. Para la conducción colectiva y participativa del estudio se conformó un equipo conductor integrado por directivos de la salud municipal, de centros de atención y referentes comunales vinculados a la atención de adolescentes.

Los objetivos de este estudio se enmarcan en los resultados de la primera fase de una investigación que busca contribuir a mejorar la implementación del Programa de la Mujer (26) y el Programa Nacional de Salud Integral de Adolescentes y Jóvenes (8). Se ha buscado caracterizar, desde la visión de los equipos de atención de salud, la ruta de acceso anticoncepción de los adolescentes y la identificación de las dificultades. Se realizó un estudio cualitativo, de carácter descriptivo, que incorporó principios de investigación acción participativa (24).

Se contó con la aprobación del Comité Ético Científico del Servicio de Salud Metropolitano Norte, las personas participantes accedieron a participar tras proceso de consentimiento informado, lo cual brindó garantías de confidencialidad.

La muestra de estudio se constituyó con 22 personas, profesionales y técnicos que participan en labores directivas, clínicas y administrativas vinculadas a la atención de adolescentes en Centros de Salud Familiar y en servicios de atención primaria de urgencia de la comuna. Se realizó muestreo por conveniencia, buscando variación máxima del discurso, y selección a través de informantes clave. La técnica utilizada fue entrevista semiestructurada, individual y grupal (cuadro 1).

**CUADRO 1.** Perfil de los entrevistados para el estudio

No.	Cargo o función	Lugar de trabajo	Sexo	Edad (años)	Profesién	Tipo de entrevista
1	Técnica en enfermería	Servicio de atención primaria de urgencia	Femenino	40	Técnica en enfermería (nivel superior)	Individual
2	Técnica en enfermería	Centro de atención primaria de la salud	Femenino	52	Técnica en enfermería (nivel superior)	Individual
3	Profesional clínica	Centro de atención primaria de la salud	Femenino	28	Matrona	Individual
4	Profesional clínica	Centro de atención primaria de la salud	Femenino	28	Piscóloga	Individual
5	Profesional clínica	Centro de atención primaria de la salud	Femenino	30	Enfermera	Individual
6	Profesional clínica	Centro de atención primaria de la salud	Femenino	27	Médica	Individual
7	Profesional clínica	Centro de atención primaria de la salud	Femenino	37	Matrona	Individual
8	Profesional clínica	Centro de atención primaria de la salud	Femenino	33	Matrona	Individual
9	Profesional clínico	Centro de atención primaria de la salud	Masculino	50	Matrón	Individual
10	Profesional clínica	Centro de atención primaria de la salud	Femenino	41	Enfermera	Individual
11	Profesional clínica	Centro de atención primaria de la salud	Femenino	58	Matrona	Individual
12	Profesional clínico	Servicio de atención primaria de urgencia	Masculino	29	Médico	Individual
13	Profesional clínico	Servicio de atención primaria de urgencia	Masculino	26	Médico	Individual
14	Jefa administrativa	Centro de atención primaria de la salud	Femenino	50	Trabajadora social	Individual
15	Jefe administrativo	Centro de atención primaria de la salud	Masculino	39	Administrador público	Individual
16	Jefe administrativo	Centro de atención primaria de la salud	Masculino	29	Psicólogo	Individual
17	Profesional	Programa comunitario de adolescentes	Femenino	31	Trabajadora social	Individual
18	Directora	Centro de atención primaria de la salud	Femenino	51	Matrona	Grupal
19	Directora	Centro de atención primaria de la salud	Femenino	51	Enfermera	Grupal
20	Directora	Centro de atención primaria de la salud	Femenino	50	Kinesióloga	Grupal
21	Directora	Centro de atención primaria de la salud	Femenino	47	Matrona	Grupal
22	Profesional de gestión	Programa adolescente	Femenino	51	Matrona	Grupal

El período de realización de entrevistas fue entre mayo y julio de 2015. Las entrevistas tuvieron lugar en centros de atención, en condiciones de privacidad y en horarios acordados con los entrevistados. Se utilizó guion temático basado en los objetivos de estudio, abierto a los énfasis y a la dinámica del diálogo establecido con cada entrevistado. Se indagaron percepciones y experiencias sobre atención y consejería de adolescentes en los centros de salud, en particular en anticoncepción, como también información y manejo sobre aspectos jurídicos y normativas sectoriales relacionadas a su atención, visión de derechos sexuales y reproductivos de los adolescentes y propuestas de mejora. Las entrevistas tuvieron una duración media de 40 minutos y fueron grabadas y transcritas en forma textual para su análisis. Fueron realizadas por las investigadoras, todas profesionales de la salud, con formación en salud pública, género, salud sexual y reproductiva y experiencia en estudios cualitativos. El análisis narrativo de contenidos guio un proceso de categorización y codificación temática, de acuerdo al mapa conceptual del estudio, con flexibilidad y sensibilidad a la emergencia de nuevas categorías. La recogida de datos y su análisis consideró tiempos e intervalos que aseguraron la circularidad y reflexividad del proceso. Los datos fueron analizados con triangulación de investigadoras en las fases de codificación e interpretación y en la determinación de saturación de la información, de cara a la respuesta a los objetivos de estudio. El proceso fue apoyado con el uso del *software* MaxQda10®.

## RESULTADOS

Se distinguieron tres momentos en los cuales los entrevistados situaron dificultades para acceso de adolescentes a anticoncepción: dificultades para la búsqueda de atención y llegada a los centros, dificultades para concretar la atención en el centro y dificultades en el proceso de atención.

### Dificultades para la búsqueda de atención y llegada a los centros

Fue consistente la percepción de baja asistencia de adolescentes a los centros, que fue crítica para los varones. Son las mujeres quienes más acuden para ser atendidas.

Aparecen en primer plano factores contextuales, con el reconocimiento de una gran vulnerabilidad social de los adolescentes en la comuna. La drogadicción, el abandono, la delincuencia y la violencia son algunas de las características destacadas, en contextos familiares marcados por la falta de tiempo de la población adulta para acompañar el desarrollo y necesidades de la población de jóvenes. Los trabajadores piensan que esto explica en parte la escasa llegada de los adolescentes, por ausencia de redes familiares y la constatación de entornos culturales distantes del sistema y sus instituciones. Estas problemáticas se concentrarían en algunos sectores muy bien identificados por los trabajadores. Algunas personas hicieron notar la presencia en las comunidades de un discurso conservador respecto de la sexualidad y escaso reconocimiento de la autonomía sexual y reproductiva de los adolescentes, en especial de las mujeres.

Los profesionales y técnicos reconocieron el inicio temprano de la actividad sexual en los adolescentes del territorio; sin embargo, señalaron que existe baja autopercepción de necesidades de atención por parte de ellos mismos. A su juicio, los adolescentes desconocen la oferta de prestaciones del centro: la entrega de anticonceptivos, control de embarazo y atención de morbilidad, donde destacan las consultas por infecciones de transmisión sexual. Mencionan la evaluación preventiva mediante la aplicación de una ficha para adolescentes, creada por el Centro Latinoamericano de Perinatología (CLAP) (27). Las consejerías no fueron visualizadas por todos, y fueron descritas en su mayoría por las matronas.

En la experiencia de los entrevistados, los adolescentes varones se aproximan con mayor frecuencia a consultas por morbilidad. En temas vinculados a la salud sexual, la mayor afluencia se produce por derivación interna, tras la detección de actividad sexual al aplicar la ficha CLAP. Coincidieron en que la principal entrada es la consulta con la matrona, por embarazo y demanda de anticonceptivos, y señalan que reciben en sus consultas principalmente a mujeres acompañadas de sus madres.

Por último, perciben que, para los adolescentes, los centros se ven distantes y burocráticos. Los horarios de atención coincidentes con la jornada escolar y los espacios poco amigables dificultan la búsqueda de atención. Perciben problemas de confidencialidad que suponen son comentados entre pares y desalientan su llegada a los centros. Todo esto está reforzado por falta de estrategias y actividades en la comunidad dirigidas a jóvenes.

Se reproducen aquí algunos *verbatims* de las entrevistas.

#### Contexto social y cultural.

“No hay comunicación, por las características de la gente de acá son casi todos súper disfuncionales. El niño va de la casa al colegio y no hay comunicación, no hay educación de parte de la familia con los niños, y muchos no están ni ahí* con los niños.” E8 Enfermera

*[no les interesan]

“Son niños muy precoces que se hacen cargo del hogar y eso hace que vayan adquiriendo actividades más precoces como consumo de drogas (…) la verdad es que el inicio de la actividad sexual es bastante más precoz de 14 años (...) E13 Matrona

*“Los adolescentes a esa edad están como bien botados, por decirlo de alguna forma, de los centros de salud y ellos tienen muchas dudas... Y no tienen a quién consultarlo porque muchas veces les da vergüenza consultarlo con los padres.”* E9 Enfermera

“También estamos en una sociedad machista, el tema de la mujer está bien castigado, desde el mismo discurso de las chiquillas** esto les genera un impedimento. Incluso al hablarlo con sus madres, es bien frecuente que las mamás tengan un discurso castigador con sus niñas”. E17 Trabajadora social

**mujeres adolescentes, mujeres jóvenes, muchachas.

#### Barreras del sistema.

“Creo que es una cobertura demasiado bajísima de los adolescentes, es como el escalón del ciclo vital perdido, porque como digo, no tienen acceso al consultorio*** si no tienen una enfermedad”. E16 Médico

***centro de salud

*“[Los adolescentes varones] Son más escasos en venir, no les gusta que los vean con la matrona … a la hermana le decimos que los traigan, le hacemos el ingreso, e igual les damos la consejería en salud sexual y reproductiva y orientamos en el uso de preservativos, pero son casos puntuales los que vienen...llegan uno o dos hombres al año”*. E1 Matrona

“No es un espacio que al adolescente le agrade venir, porque está la vecina y acá el tema del copuchenteo**** es bien potente entre las vecinas ahí, entonces yo creo que les da vergüenza”. E3 Psicóloga

****chisme, habladuría.

*“Nosotros no salimos a buscarlos, no salimos a su encuentro, no nos insertamos en su mundo. Entonces, ¿cómo esperamos que ellos adhieran a un sistema? Si nosotros no los reconocemos ni nos validamos en su ambiente tampoco”*. EG1 Directivas

### Dificultades para concretar una atención en el centro de salud

Una vez en el centro, existen barreras vinculadas a procedimientos administrativos previos a la atención profesional, donde la solicitud de documentación, la indagación del motivo de consulta y la necesidad de compañía de un adulto son hitos que complejizan y dificultan el acceso. Los relatos muestran que estas situaciones dejan en el camino a adolescentes que, habiendo llegado al centro de salud, desisten frente a estos obstáculos.

En primer lugar, se les solicita la presentación de documentos que acrediten su inscripción como beneficiario del centro. Muchas veces refieren que los adolescentes desconocen si su familia está inscrita y no tienen posibilidad de traer documentos de manera autónoma. Declaran que en los centros se les exige la compañía de un adulto para la atención, sobre todo los menores de 14 años, motivado por el resguardo legal de los profesionales. En ocasiones, el personal técnico se ofrece a acompañarlos para sortear esta dificultad. Profesionales que ya han establecido vínculo por atenciones previas declaran experiencias con adolescentes que luego les buscan directamente en sus consultas.

El personal administrativo señala que, al ingreso, deben decidir a qué tipo de atención le corresponde, para lo cual indagan el motivo de consulta. Los relatos evidenciaron dificultades de comunicación con los adolescentes y dejan ver también interpretaciones que develan prejuicios respecto de su sexualidad. Estas interacciones son percibidas por profesionales y técnicos como momentos donde los adolescentes sienten vulnerada su confidencialidad y se sienten expuestos, ya que con frecuencia ocurre en lugares abiertos, contiguos a salas de espera, en presencia de otras personas.

Se reproducen aquí algunos *verbatims* de las entrevistas.

#### Solicitud de documentación y horario de atención.

“A los adolescentes se les pide los mismos requisitos que a todo el mundo: certificado de residencia o domicilio, cédula de identidad, y que estén inscritos en FONASA*, son los requisitos que te obliga el sistema, eso es así”. EG1 Directivas.

*Fondo Nacional de Salud

*“Los adolescentes no vendrán a atenderse con el médico o con la matrona, si para pedir una hora con estos profesionales se tienen que levantar a las siete y media de la mañana y presentar todos los documentos que se les exige, entonces mejor prefieren no acudir al centro”*. E4 Profesional administrativo

#### Exposición y falta de confidencialidad.

“Es que generalmente las adolescentes cuentan primero su problema a la persona administrativa que da la hora de atención, diciendo, por ejemplo— ayer tuve un problema, tuve relaciones con mi pololo** y no me cuidé, entonces después de manifestar su necesidad se da aviso a la matrona”. E4 Profesional administrativo

**enamorado, prometido, pretendiente, pareja, novio.

*“Me imagino que algunos adolescentes llegan directo a atenderse con los profesionales también, porque hacerlos pasar a contar su problema al mesón, y que en el mesón les digan, vaya a ver a las técnicas de enfermería y después de eso, van a ser atendidos por profesionales... en todo ese recorrido, se ha manoseado mucho su intimidad”*. E3 Psicóloga

*“Nosotros tenemos en las fichas clínicas a las mamás, al papá, al hermano y entonces ellos [adolescentes] temen que se puedan encontrar con sus familiares y con el vecino”*. E13 Matrona

*“Yo creo que les da vergüenza venir a los 13 años, como les pasa a estos adolescentes [que me pidieron directamente condones]: No, es que dile tú; No, tú… - es porque tienen vergüenza, no se atreven”*. E7 Técnica de enfermería

#### Compañía de un adulto en la recepción.

*“Dije, ¿pero qué edad tienes?, diecisiete años, yo le dije, pero tú tienes que venir con un adulto a pedir hora, porque yo no te puedo dejar pasar al Médico, —[era] un menor de dieciocho años—, porque indica la ley que no puede pasar ningún menor de edad, por el tema de la confidencialidad, de que se pueda acusar al Médico o al profesional de cosas, pero ese muchacho después no vino y no me dijo nada”*. E4 Profesional administrativo

*“Generalmente nosotros prohibimos y rechazamos las personas que vengan solas a no ser que te plantee el tema y tú hablas directamente con la matrona y es la matrona la que toma la decisión y dice vamos hacer esto y esto, tenemos que contarles a los papás”*. E4 Profesional administrativo

*“Es que no vienen solos [adolescentes], vienen acompañados, a no ser que tengan 18 o 19 años, ahí vienen solos”*. E9 Enfermera

**FIGURA 1.**
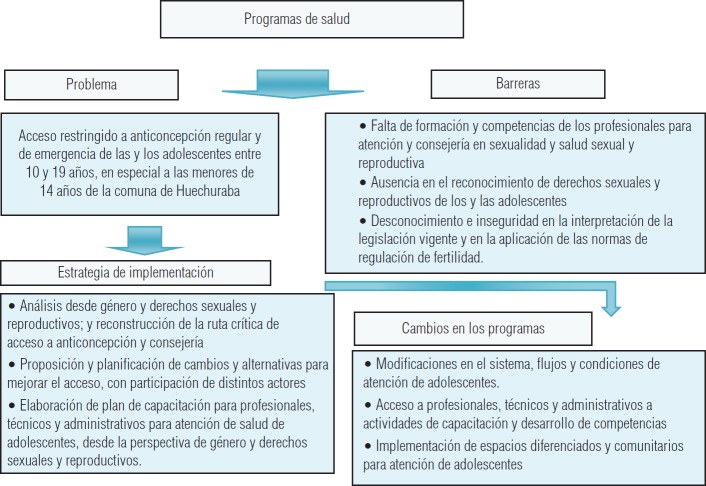
Flujograma de problemas, barreras diagnosticadas y estrategias para el mejoramiento de programas en el marco del estudio según el modelo iPIER.

### Dificultades en el proceso de atención

Los relatos de los entrevistados respecto de experiencias de atención de adolescentes muestran heterogeneidad en conductas profesionales. Hay distintas posturas, según sus conocimientos y valores, por lo general en ausencia de enfoque de género y derechos (figura 1). En los discursos predomina una perspectiva Técnica y biomédica, donde el resguardo de la actuación profesional resulta muy relevante. Aunque al referirse a contextos en los que viven la población de jóvenes se señalan problemas sociales que dificultan el acceso, no hay relato sobre acciones consensuadas para su abordaje.

Los trabajadores reconocieron la falta de conocimiento y seguridad en la interpretación de normativas sanitarias y leyes que fundamentan y regulan el acceso de los/las adolescentes a las prestaciones que ofrece el centro (figura 1). Esto se vuelve más crítico a menor edad de las jóvenes. La búsqueda de anticonceptivos en menores de 14 años es una de las situaciones donde profesionales clínicos manifestaron mayor inseguridad, por el hecho de que la ley estipula la realización de una denuncia por sospecha de abuso sexual. También para el personal técnico y administrativo estas atenciones resultaban problemáticas cuando los adolescentes acudían sin compañía de una persona adulta.

La aplicación de la ficha CLAP se presenta como un facilitador de acceso a la atención; sin embargo, aunque permite la identificación de adolescentes sexualmente activos, no parece garantizar el seguimiento y el acceso a métodos anticonceptivos.

Se reproducen aquí algunos *verbatims* de las entrevistas.

***Falta de formación en derechos sexuales y reproductivos***

*“¿Derechos sexuales y reproductivos?, los desconozco … he visto que se dan charlas de educación sexual por parte de técnicos y enfermeras, pero en la consulta con el poco tiempo que tenemos cuando uno tiene a un adolescente uno siempre trata de abordar ese tema…”*. E14 Médico

#### Desconocimiento y heterogeneidad en el abordaje.

“Con la pastilla del día después [anticoncepción de emergencia] teníamos un problema que no sabíamos si poder darla o no a una adolescente porque venía con la nuera… Y qué tuvimos que hacer…buscar en *internet, guglearlo. Y ahí descubrimos que había que entregarlo de todas maneras”*. E5 Técnica de Enfermería

*“Nosotros tenemos la obligación de dársela [anticoncepción de emergencia], previa educación, no nos podemos negar. Pero eso es también un desconocimiento, porque ellas piensan que nosotros vamos a llamar a los papás porque están requiriendo la pastilla y no es así”*. E8 Enfermera

#### Denuncia en menores de 14 años sexualmente activas.

*“Si pues, siempre se tiene que hacer la denuncia porque quizás sea abusada en la casa, o bien si tiene un pololo de 14. La fiscalía tiene que investigar, es una manera de protegerlas”*. E6 Matrona

#### Compañía de un adulto en la atención clínica.

*“Más que nada [vienen] mujeres solas y con amigas, una les da la consejería y las va orientando a cualquier método que ella elija. En menores de 15 les decimos que venga con un adulto mayor responsable, abuelita, mamá”*. E1 Matrona

*“[Una adolescente menor de 14 años] Si viene así abiertamente es porque necesita método anticonceptivo la derivamos a la matrona, y yo creo que la matrona hace pasar a un técnico como para que quede registro de que no viene sola, porque nosotros no podemos atender menores de edad sin un adulto responsable..*.*porque así dice la ley...al momento del examen físico, hago pasar a la mamá a al acompañante, porque no le puedo sacar la ropa si no están los papás*. E9 Enfermera

## DISCUSIÓN

Los resultados muestran la inexistencia de una ruta clara y consensuada de acceso a atención y consejería en materia de salud sexual y reproductiva para los adolescentes de la comuna de Huechuraba. Los obstáculos culturales, de información y de carácter administrativo limitan y desincentivan el acceso, lo cual, según los testimonios, afecta en especial a la población joven más vulnerable, que no está en condiciones de responder con éxito a solicitudes administrativas y de acompañamiento. En este contexto, los discursos de los trabajadores resultan contradictorios cuando señalan que los adolescentes no acuden porque no perciben necesidades, no tienen información sobre prestaciones, o por condiciones de vulnerabilidad, y al mismo tiempo reconocen participar de acciones que claramente obstaculizan el acceso a la atención de los adolescentes. Esto es crítico en el caso de mujeres menores de 14 años, donde se impide su ingreso si no van acompañadas de un adulto; en esas condiciones, también su atención clínica podría ser rechazada por profesionales. Resulta paradojal que luego se afirme que las adolescentes no acuden solas de manera espontánea.

La falta de conocimientos sobre normas de fertilidad, legislación vigente y su adecuada interpretación, así como el manejo de enfoque de género y derechos sobre salud sexual y reproductiva, evidencian debilidades de los profesionales y el personal administrativo en competencias fundamentales para trabajar con adolescentes. Los resultados levantados por este estudio confluyen con otros diagnósticos y estudios nacionales e internacionales, respecto de dificultades de acceso a anticoncepción de los adolescentes más vulnerables (28), en particular las menores de 14 años, por inseguridad y falta de información respecto de normativas y marcos legales relacionados a su atención (29). Las falencias en la formación profesional respecto a la confidencialidad, salud sexual y reproductiva, género y derechos sexuales y reproductivos también son observadas como una dificultad relevante en otros contextos (12-14, 16, 22, 30).

La información sobre lo que el centro de salud puede ofrecer a los adolescentes resulta relevante en la fase previa a la llegada, las acciones de difusión en la comunidad podrían contribuir a hacer el centro menos distante, así como el establecimiento de lugares de atención en otro tipo de espacios. Parra y Domínguez (31) enfatizan la intensificación de actividades de difusión de información sobre salud sexual y reproductiva, y recomiendan que las actividades se realicen en el entorno cercano, en centros educativos y en centros comunitarios.

La experiencia de este proyecto hizo posible que los resultados del estudio contribuyeran, junto a otras iniciativas comunales, a conformar una mesa de trabajo intersectorial para la construcción e implementación de una política comunal consensuada para el trabajo con adolescentes del territorio. El acceso a la atención de adolescentes constituyó una prioridad en el Plan de Salud de la Comuna para el año 2016 y en la actualidad se impulsa la creación de un espacio diferenciado para adolescentes de la comuna, fuera de las dependencias de los centros de salud y de carácter intersectorial e integral, donde uno de los focos es la atención promocional y preventiva en salud sexual y reproductiva. Dentro de otras acciones se capacitó en materia de género, derechos sexuales y reproductivos en un curso integrado por profesionales y personal técnico y administrativo y se trabaja en el diseño participativo de un protocolo de ingreso para la atención de adolescentes y entrega de anticoncepción. Para facilitar el acceso a los preservativos, se han instalado dispensadores gratuitos en cada uno de los establecimientos de salud, bibliotecas y oficinas de la juventud, ubicados en lugares de fácil concurrencia de adolescentes. Para completar este diagnóstico está en curso una segunda fase de estudio, en la que se releva información desde la experiencia de los propios adolescentes de la comuna, cuyos resultados se espera incidan en las definiciones estratégicas y en mayor incorporación de adolescentes en espacios de participación.

## Conclusiones

Se encontró una inadecuada respuesta institucional frente a los cambios culturales y sociales detectados por los equipos, donde las necesidades en salud sexual de los adolescentes parecen subvaloradas e incomprendidas. Se evidenció ausencia de reflexión para el acceso efectivo a anticoncepción y consejería de adolescentes, en particular en mujeres menores de 14 años y varones. Las condiciones actuales atentan contra la llegada, visibilidad y respuesta a las necesidades en salud sexual y reproductiva de los adolescentes, como al desarrollo de acciones promocionales y preventivas. Se requieren puertas abiertas, mayor conexión con la comunidad y múltiples caminos. Junto con esfuerzos en la formación, que deben dirigirse a todo el personal de los centros de salud, se necesitan espacios de reflexión que permitan la generación de respuestas articuladas frente a los problemas detectados. Resulta necesaria y urgente la investigación con los propios adolescentes, incorporando sus visiones y realidades, y fijando como horizonte inmediato y futuro el acceso efectivo, en un marco de respeto a su autonomía y derechos sexuales y reproductivos.

### Financiamiento.

Este trabajo fue financiado por la Alianza para la Investigación en Políticas y Sistemas de Salud (AHPSR), de la Organización Mundial de la Salud (OMS) y la Organización Panamericana de la Salud (OPS). La OPS brindó cooperación técnica para el desarrollo de este proyecto. En el contexto del programa iPIER, el Instituto de Efectividad Clínica y Sanitaria (IECS) brindó asistencia técnica para el desarrollo del protocolo, la ejecución y publicación del proyecto.

### Declaración.

Las opiniones expresadas aquí son responsabilidad de los autores y no reflejan necesariamente el criterio ni la política de la *RPSP/PAJPH* y/o la Organización Panamericana de la Salud/Organización Mundial de la Salud.
